# Comparing mortality between cancer and non-cancer critically ill patients: a propensity score matched analysis

**DOI:** 10.1186/2197-425X-3-S1-A150

**Published:** 2015-10-01

**Authors:** R Rosa, B Duso, M Mattioni, MD Rosa, AM Ascoli, J Maccari, T Tonietto, JH Barth, P Morandi, F Dexheimer, W Rutzen, L Madeira, L Tagliari, RPD Oliveira, C Teixeira

**Affiliations:** Moinhos de Vento Hospital, Porto Alegre, Brazil

## Introduction

Intensive care unit (ICU) admission may be required in cancer patients for management of acute illnessess associated with underlying malignancy or complications of cancer therapy. The higher mortality rates found in this population, in comparison to non-cancer critically ill patients, may be attributable to confounding bias due to a higher incidence of multiple organic dysfunction in those patiens with malignant neoplasms; however data regarding this hypothesis are scarce.

## Objectives

To compare the crude and propensity score matched mortality rates between cancer and non-cancer adult patients admited to a mixed medical-surgical ICU.

## Methods

We conducted a retrospective analysis of a comprehensive longitudinal ICU database from a tertiary referral hospital in Southern Brazil. All adult patients who were admitted to the ICU from January 2001 to December 2008 were evaluated. Crude and propensity score adjusted all cause 30-day mortality rates of critically ill cancer patients were compared with those of critically ill patients without cancer.

## Results

A total of 4221 patients were evaluated. The overral population 30-day mortality was 12.2 %. In a Cox regression analysis, crude mortality rates were higher among cancer patients (HR, 1.93; 95%CI, 1.61-2.31). However, after adjusting for propensity score, the 30-day mortality rates of cancer and non-cancer patients were similar (HR, 1.15; 95% CI, 0.88-1.50).

## Conclusions

The present study showed that the higher crude 30-day mortality found in ICU cancer patients, when comparable to non-cancer patients, was a result of confounding. The propensity score matched analysis showed no evidence of excess of mortality in critically ill patients due to cancer diagnosis.
